# Patients with overlapping dermatomyositis and psoriasis: an experience from a tertiary center and review of the literature

**DOI:** 10.1186/s42358-025-00454-7

**Published:** 2025-05-07

**Authors:** Beatriz Westphalen Pomianoski, Samuel Katsuyuki Shinjo

**Affiliations:** Division of Rheumatology, Faculdade de Medicina FMUSP, Universidade de São Paulo, Av. Dr. Arnaldo, 455, 3º andar, sala 3184 - Cerqueira César, São Paulo, SP, BR CEP: 01246-903 Brazil

**Keywords:** Autoimmune disease, Dermatomyositis, Myositis, Overlapping syndrome, Psoriasis

## Abstract

**Background:**

The coexistence of dermatomyositis (DM)/clinically amyopathic DM (CADM) and psoriasis has been infrequently documented in the literature. Consequently, this study aimed to analyze this entity from our tertiary center and review the relevant literature.

**Methods:**

This retrospective observational cross-sectional study and case series included patients with DM/CADM and psoriasis between 1998 and 2024. A literature review was also conducted.

**Results:**

Nine of 331 patients with DM (*n* = 265)/CADM (*n* = 66) had psoriasis; six were female, and all were of white ethnicity. The median age at DM diagnosis was 38 years (range: 18–78), and at psoriasis diagnosis was 43 years (range: 18–81), with a median interval of four years between diagnoses. The follow-up revealed that six patients were discharged, two died, and one continued follow-up. The primary comorbidities included systemic arterial hypertension (*n* = 3) and diabetes mellitus (*n* = 3). Four patients presented with varicella zoster (*n* = 1) or pulmonary tuberculosis (*n* = 3). Regarding the literature review, 15 articles reported a total of 17 cases of overlapping DM/CADM and psoriasis. However, variability was observed in the DM/CADM diagnostic criteria. The mean age at DM diagnosis in the literature was 32.3 years (range: 2–59), whereas for psoriasis, it was 31 (7–63) years. Female patients were predominant.

**Conclusion:**

This investigation identified the coexistence of DMPs, with a median age of 38 years for DM and 43 years for psoriasis. The variability in the diagnostic criteria underscores the necessity for standardized approaches to enhance patient management.

## Introduction

Dermatomyositis (DM) is an idiopathic inflammatory myopathy or systemic autoimmune myopathy characterized by cutaneous manifestations and skeletal muscle weakness [1, 2]. Classical cutaneous lesions include heliotrope rash and Gottron’s papule/sign, whereas secondary manifestations comprise “shawl” sign, “V-neck” sign, “holster” sign, “flagellate” erythema, periungual hypertrophy and hyperemia, and calcinosis [3].

Psoriasis is a prevalent, non-communicable, chronic inflammatory dermatological condition that presents with diverse cutaneous manifestations. It can manifest in various forms, including symmetrical erythematous and scaly plaques, generalized or focal pustules on the palmar and plantar surfaces, pruritus, pain, nail alterations, joint stiffness, and edema [4, 5]. Psoriasis vulgaris represents the most common subtype [4].

The association between DM and psoriasis (DMPs) has been infrequently described in the literature, with documentation limited to a small number of case reports [6–20]. The majority of these reports did not include analyses of specific antibodies for DM [7, 8, 10, 11, 17, 20], or did not employ specific DM classification criteria [9, 13]. Furthermore, the existing literature on DMPs exhibits significant gaps and a paucity of information regarding diagnostic data, medication regimens, and disease status [10, 12, 14, 19]. Consequently, this study aimed to conduct a comprehensive descriptive analysis (encompassing demographic, clinical, laboratory, therapeutic, and evolutionary aspects) of patients with DMPs in a Brazilian tertiary center. Additionally, we performed a review of the relevant literature.

## Materials and methods

This retrospective, cross-sectional, single-center study and a series cases included patients with DMPs from our tertiary center between 1998 and 2024.

DM was defined according to the Bohan and Peter [21] and European League Against Rheumatism / American College of Rheumatology (EULAR/ACR) 2017 classification criteria for idiopathic inflammatory myopathies [22]. CADM was also defined according to Gerami et al.’s criteria [23]. The diagnosis of psoriasis was based on a clinical assessment performed by dermatologists; analysis of the shape, color, region, and other characteristics of the dermatological lesions; and/or complemented by a biopsy examination, in addition to the assessment of systemic symptoms.

Patients with questionable DM, DMPs, psoriasis, or incomplete data were excluded from the study.

Pre-parameterized and pre-standardized data were collected from the electronic medical records:

- Demographic data: Age at onset of illness, sex, ethnicity, diagnostic data, classification of the type of DM, and psoriasis;
- Clinical data: Muscle weakness, presence of Gottron’s papules, heliotrope rash, calcinosis, Raynaud’s characteristics, “mechanic’s hands,” etc. The characteristics of psoriatic lesions and their anatomical distributions were determined;
- Data from complementary and laboratory tests: autoantibodies, initial creatine phosphokinase (CPK), aldolase, muscle biopsy, and electroneuromyography. The autoantibodies were identified using a commercial line immunoassay, EUROLINE Autoimmune Inflammatory Myopathies 16 Ag (IgG) (Euroimmun, Lübeck, Germany), in which only strong reactions (+++) assessed by two independent readers were considered positive, according to a previous study [24].
- Data from the first and last consultation, follow-up, death, drug treatment carried out during follow-up and last reported treatment, and disease activity status, which were divided into three different responses: complete clinical response (CCR), use of immunosuppresses without the use of glucocorticoids for more than six months; remission, when no treatment for more than six months; disease activity, when there are clinical (cutaneous and/or objective muscle limb weakness) and laboratory activities (increase in serum levels of muscle enzymes).
- Comorbidities such as diabetes mellitus, dyslipidemia, systemic arterial hypertension, acute myocardial infarction, heart failure, varicella-zoster virus, and tuberculosis.

### Statistical analysis

The Shapiro-Wilk test was employed to assess the distribution of each parameter. The results were expressed as mean ± standard deviation (SD) or median (minimum - maximum) for continuous variables, whereas categorical variables were presented as frequency (%). The analyses were performed using SPSS software (version 15.0, Chicago, IL, USA).

### Literature review

#### Search strategy

We searched the National Library of Medicine (PubMed), SciELO, and EMBASE platforms from inception to March 2024 with the following search terms: “dermatomyositis” AND “psoriasis.”

#### Eligibility and exclusion criteria

Only case reports or studies on DMPs were included. Review articles, systematic reviews, meta-analyses, qualitative clinical studies, duplicates, and conference manuscripts were excluded. There were no limitations to the language used in the articles. The selection process included patients of all ethnicities and ages. Patients with overlapping disease mimicry were excluded from the study.

#### Manuscript selection criteria

The selection was independently performed in two stages by two researchers: (*i*) selection of articles based on abstract DMPs and (*ii*) those selected from the first stage were fully screened and selected.

## Results

### Cases from the present study

A total of 331 patients were evaluated: 265 with DM and 66 with CADM between 1998 and 2024.

Nine patients also had psoriasis: four with DM (1.5%) and five with CADM (7.6%). Six patients were female and all were of white ethnicity (Table 1).

**Table 1:** Patients with overlapping dermatomyositis and psoriasis from the present study and literature

Authors [Ref]	Year	Sex	Origin or ethnicity	Type of DM	Age at DM diagnosis (years)	Type of psoriasis	Age at psoriasis diagnosis (years)	Interval between the diseases (years)
Current study	2024	M	White	DM	78	Vulgar psoriasis	81	3
		M	White	CADM	39	Vulgar psoriasis	43	4
		F	White	CADM	48	Vulgar psoriasis	50	2
		F	White	DM	18	Vulgar psoriasis	19	1
		F	White	DM	28	Vulgar psoriasis	42	14
		M	White	CADM	19	Vulgar psoriasis	27	8
		F	White	CADM	24	Psoriasis	36	12
		F	White	CADM	38	Psoriasis	47	7
		F	White	DM	60	Psoriasis	62	2
Xu et al. [7]	2023	F	Asian	DM	41	Psoriasis	14	27
Chu et al. [6]	2022	M	Chinese	DM	63	Psoriasis	53	10
Perna et al. [8]	2022	F	White	JDM	N/A	Psoriasis	N/A	N/A
Schreiber et al. [9]	2021	F	Hispanic	DM	45	Psoriatic arthritis	N/A	N/A
Markovic et al. [10]	2019	F	White	JDM	5	Psoriasis	5	0
Xing et al. [11]	2018	M	Chinese	JDM	15	Vulgar psoriasis	21	6
Kato et al. [12]	2017	F	N/A	DM	30	Psoriasis	35	5
Inkeles et al. [13]	2017	F	N/A	CADM	45	Psoriasis	N/A	N/A
Montoya et al. [14]	2017	M	N/A	CAJDM	15	Psoriasis	20	5
Akiyama et al. [15]	2016	F	Japanese	DM	52	Psoriasis	60	8
Dicaro et al. [16]	2014	F	Brazilian	DM	37	Psoriasis	30	7
Kim et al. [17]	2011	F	White	JDM	8	Psoriasis	18	9
		F	Indigenous	JDM	8	Psoriatic arthritis	7	< 1
		F	White	JDM	2	Psoriasis	N/A	N/A
Machado et al. [18]	2010	M	Brazilian	DM	41	Vulgar psoriasis	45	4
Gran et al. [19]	2009	M	White	DM	50	Psoriasis	N/A	N/A
Pavlovic et al. [20]	2004	M	White	DM	59	Psoriasis	63	4

CADM: clinically amyopathic dermatomyositis; CAJDM: clinically amyopathic juvenile dermatomyositis; DM: dermatomyositis; F: female; JDM: juvenile dermatomyositis; M: male; N/A: not available

The median age at the time of DM diagnosis was 38 (18–78) years, whereas the median age at psoriasis diagnosis was 43 (18–81) years. Therefore, the median interval between disease diagnoses was four years. The general patient characteristics are shown in Table 2.

**Table 2:** General features of dermatomyositis from the present study and literature

Authors [Ref]	Diagnosis	Antibodies	Cutaneous	Muscle	Dysphagia	Lung	Joint	Previous treatment	Current treatment	Current disease status
Current study	Bohan & Peter, and EULAR/ACR	PM/Scl(+)	G, H, MH	MW, ENMG, Lab	Yes	Yes	-	AZA, MTX	MMF 1 g/day	CCR
		MDA-5(+)	G, H, CA, RP	-	-	-	Yes	AZA	MTX 25 mg/week	CCR
		PM/Scl(+)	G, H, RP, MH	MW, Lab	Yes	Yes	Yes	AZA, CP, MTX	MMF 1.44 g/day	CCR
		All negative*	G, H, CA	MW, ENMG, Bx, Lab	Yes	-	Yes	AZA, CP	MTX 15 mg/week, MMF 1 g/day	Remission
		Ro-52(+), PM/Scl(+)	G, H, RP, MH	MW, ENMG, Bx	Yes	Yes	Yes	AZA, MTX	-	Remission
		MDA-5(+)	G, H	-	-	Yes	Yes	AZA, CP. MMF, RITUX	MTX 20 mg/week	CCR
		All negative*	G, H	MW, ENMG, Bx, Lab	Yes	-	Yes	MTX	Aza 150 mg/day GC 40 mg/day	Remission
		MDA-5(+)	G, H	MW	-	Yes	-	AZA, CP, MMF, MTX, RITUX	-	Remission
		Jo(+)	G, RP, MH	MW, ENMG, Bx,	Yes	Yes	Yes	-	MMF 2 g/day, MTX 12.5 mg/week	Remission
Chu et al. [6]	Bohan & Peter Cut, Musc	MSA(-)	G, H, VS, SP	MW, Bx, ENMG	-	-	-	MTX, MP, HCQ	MP 8 mg/day, HCQ 200 mg/day, MTX 5 mg/week	Activity
Xu et al. [7]	Cut, Musc	ANA(+)	H, VS, SS, PRSL	MW, Bx	-	Yes	Yes	ADA	Upadacitinib 15 mg/day	Controlled
Perna et al. [8]	Cut, Musc	N/A	G, H, VS, VEP	MW, Bx	-	-	Yes	MTX	MTX	Activity
Schreiber et al. [9]	Cut, Musc	Jo(+), AMA(+), AML(+), ANA(-), CCP(-)	MH	MW, MRI, Bx	-	-	Yes	AZA, MTX, MMF	MTX 20 mg/week	Controlled
Markovic et al. [10]	EULAR/ACR	ANA(+)	G, H, SP, VEP	MW, Bx	-	-	-	N/A	N/A	Controlled
Xing et al. [11]	Cut, Musc	N/A	G, H	MW, ENMG, Bx	-	Yes	-	MTX, CyA	MTX 7.5 mg/week	Controlled
Kato et al. [12]	Cut, Musc, LA, Ab	ANA(+), Jo(-)	G, H, RP, MH, VS	MW, Bx	-	Yes	Yes	MP	N/A	Remission
Inkeles et al. [13]	Cut, Ab	ANA(+), sDNA(-), SSA(-), SSB(-), Sm(-), RNP(-), aCL(-), Scl70(-), ACA(-)	nonspecific interface dermatitis with perivascular inflammation	-	-	-	-	AZA, MTX, MMF, CyA, RTX, HCQ, UST	CyA 5 mg/kg/day	Remission
Montoya et al. [14]	Cut, Musc	ANA(-), Jo(-), Mi2(-)	G, SP, PE	Bx, ENMG	-	-	-	RTX, MP	UST 45 mg/12week	CCR
Akiyama et al. [15]	Cut, Musc, Ab	ANA(+), SSA(+), TIF-1 g(+)	G, H, SS	MW, Bx	-	-	-	MTX, CyA, MMF, GC	MTX 6 mg/kg	CCR
Dicaro et al. [16]	Cut, Musc, LA	ANA(-), Scl70(-)	G, SP	Bx	-	-	-	MTX, ADA	N/A	CCR
Kim et al. [17]	Cut, Musc	N/A	G, H	MW, Bx, MRI	-	-	-	MP	MTX	CCR
	Cut, Musc, LA	N/A	G, H	MW	-	-	-	MTX, MMF, CyA	MMF 20 mg/kg, CP (80-100 mg/mL	CCR
	Cut	N/A	G, H	MW, MRI, Bx	-	-	-	MTX	MTX 25 mg/m2/week	CCR
Machado et al. [18]	Bohan & Peter	ANA(+), ENA(-)	Typical rash	MW, Bx	-	-	Yes	CyA	CYC 150 mg/day	Activity
Gran et al. [19]	Cut, Musc	ANA(-), MSA(-)	G, H, VS	MW, MRI	Yes	-	Yes	AZA	MP 1 g	Activity
Pavlovic et al. [20]	Cut, Musc, LA	N/A	G, SP	MW, Bx. ENMG	-	-	-	AZA, MP	Aza 2 mg/kg/day	Remission

* Anti-Jo-1, -EJ, -OJ, -PL-7, -PL-12, -MDA-5, -NXP-2, -TIF1-Ⓒ, -SAE, - SRP, -Mi-2, -Ro-52, -Ku, -PM/Scl

ACA: anticentromere; Ab: antibody; aCL: anticardiolipin; ADA: adalimumab; ANA: antinuclear antibody; AZA: azathioprine; Bx: biopsy; CA: calcinosis; CCR: complete clinical response; CyA: cyclosporine; CYC: cyclophosphamide; Cut: cutaneous; ENMG: electroneuromyography; G: Gottron; GC: glucocorticoid; H: heliotrope rash; HCQ: hydroxychloroquine; Hk: Hyperkeratosis; LA: laboratory analysis. MH: mechanic’s hand; MMF: mycophenolate mofetil; MP: methylprednisolone; MRI: magnetic resonance images; MSA: myositis-specific antibodies; MTX: methotrexate; Musc: muscle; MW: muscle weakness; N/A: not available; PE: periungual erythema; PRSL: purplish-red skin lesions; RITUX: rituximab; RP: Raynaud’s phenomenon; SP: scaly plaques; SS: shawl sign; VEP: violaceous erythematous patches; VS: V-neck sign; UST: ustekinumab

Regarding laboratory analysis, the median serum level of CPK was 210 (59-2821) U/L, whereas the median serum level of aldolase was 10 (4.3–49.9) U/L. The distribution of myositis-specific and myositis-associated autoantibodies is shown in Table 2.

All patients in our study received methotrexate, eight received azathioprine, and six received mycophenolate mofetil.

Regarding psoriasis, 67% of the patients had psoriasis vulgaris, and all patients were treated with glucocorticoids. The general patient characteristics are shown in Table 3.

**Table 3:** General features of psoriasis from the present study and literature

Authors [Ref]	Type	Diagnosis	Cutaneous	Treatment	Current disease status
Current study	Psoriasis vulgaris	Cut, Bx	Extensor area of proximal interphalangeal joints	GC, Calcipotriol	Controlled
	Psoriasis vulgaris	Cut, Bx	Extensor area of elbows, knees, and hands*	GC	Controlled
	Psoriasis vulgaris	Cut, Bx	Extensor area of elbows, knees, and hands	GC	Controlled
	Psoriasis vulgaris	Cut, Bx	Extensor area of elbows, knees, and hands	GC	Controlled
	Psoriasis vulgaris	Cut, Bx	Palmar areas, and extensor area of knees	GC, Calcipotriol	Controlled
	Psoriasis vulgaris	Cut, Bx	Extensor area of elbows, knees, and hands	Tacrolimus, Primecolimus, GC	Controlled
	Psoriasis	Cut, Bx	Extensor area of legs and forearms, and scalp	GC	Controlled
	Psoriasis	Cut, Bx	Extensor area of elbows, knees, and hands	GC	Controlled
	Psoriasis	Cut, Bx	Extensor area of elbows**	GC	Partially controlled
Chu et al. [6]	Psoriasis	Cut	N/A	GC	Controlled
Xu et al. [7]	Psoriasis	Cut, Bx	N/A	GC	Controlled
Perna et al. [8]	Psoriasis	Cut, Bx	N/A	GC/TNF/anti–IL-12/23/ ADA, ETA, MTX, SEC	Controlled
Schreiber et al. [9]	Psoriatic arthritis	Cut	Swelling of proximal interphalangeal and distal interphalangeal joints	GC	Controlled
Markovic et al. [10]	Psoriasis	Cut	Erythematous plaques with scaling on the cheeks and nose	N/A	N/A
Xing et al. [11]	Psoriasis vulgaris	Cut, Bx	Pruritic erythema on the face, neck and back	Fluticasone, calcipotriol	Controlled
Kato et al. [12]	Psoriasis	Cut	Erythematous plaques on the scalp and elbows, knees	N/A	N/A
Inkeles et al. [13]	Psoriasis	Cut	N/A	CyA	Controlled
Montoya et al. [14]	Erythrodermic psoriasis	Cut, Bx	Erythematous plaques on dorsal and lumbosacral, lumbar region, anterior part of the chest and extensor surfaces of the upper and lower limbs	N/A	Controlled
Akiyama et al. [15]	Psoriasis	Cut, Bx	Erythematous plaques on the face, neck, back and elbows	GC, calcitriol	Controlled
Dicaro et al. [16]	Psoriasis	Cut	Erythematous plaques on the back	ADA	Controlled
Kim et al. [17]	Psoriasis	Cut, Bx	Erythematous plaques on the back	MTX, GC	Controlled
	Psoriatic arthritis	Cut, Bx	typical lesions of psoriatic arthritis	GC, MTX	Controlled
	Psoriasis	Cut, Bx	Erythematous plaques on the scalp, face, chest, back and legs	GC	Controlled
Machado et al. [18]	Psoriasis vulgaris	Cut, Bx	N/A	GC	Controlled
Gran et al. [19]	Psoriasis	Cut	N/A	Acitretin	N/A
Pavlovic et al. [20]	Psoriasis	Cut, Bx	Erythematous plaques	GC, keratolytics	Controlled

ADA: adalimumab; Bx: biopsy; Cut: cutaneous; CyA: cyclosporine; ETA: etanercept; GC: glucocorticoid; IL: interleukin; MTX: methotrexate; N/A: not applicable; SEC: sequikinumab; TNF: tumour necrosis factor

**Fig. 1:**
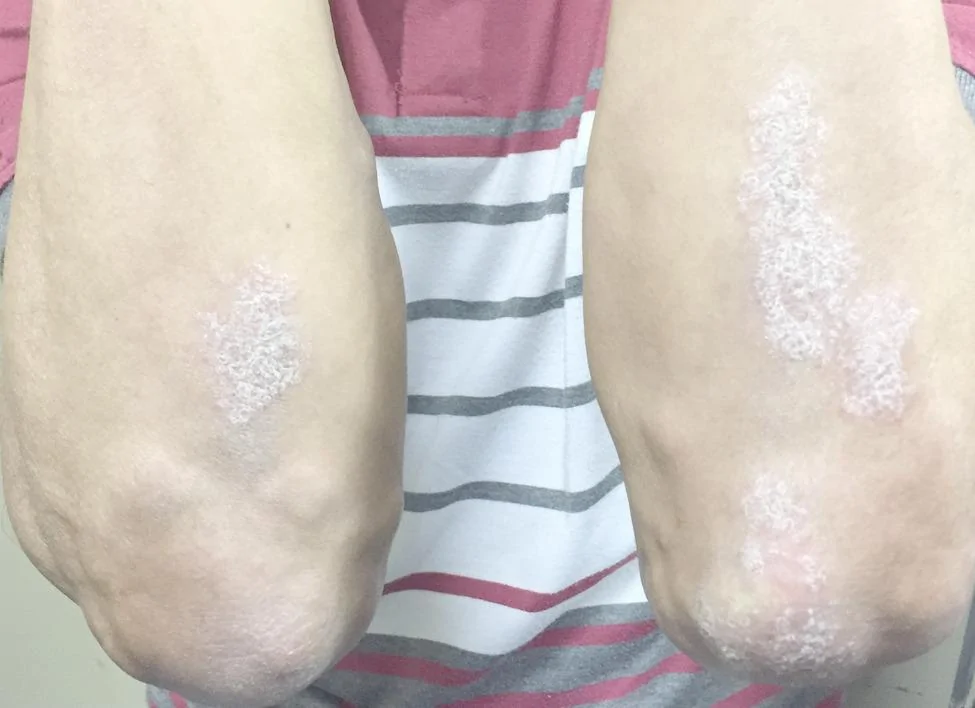
A male patient with clinically amyopathic dermatomyositis (remission) and psoriasis in elbows

Figure 1 shows a 34 years-old male patient with CADM who was evaluated for disease remission. However, after four years, psoriasis was evaluated in both elbows.

The average and median follow-up periods of the patients were 120 (30–198) months. Six patients were discharged, two died, and one patient continued treatment.

Regarding the presence of comorbidities and infections, three patients had systemic arterial hypertension, three had diabetes mellitus, three had varicella-zoster virus, two had dyslipidemia, and six had other comorbidities (atrioventricular block, acute myocardial infarction, focal segmental glomerulosclerosis, heart failure, osteoarthritis, pulmonary fibrosis, and pulmonary tuberculosis).

### Literature review

**Fig. 2:**
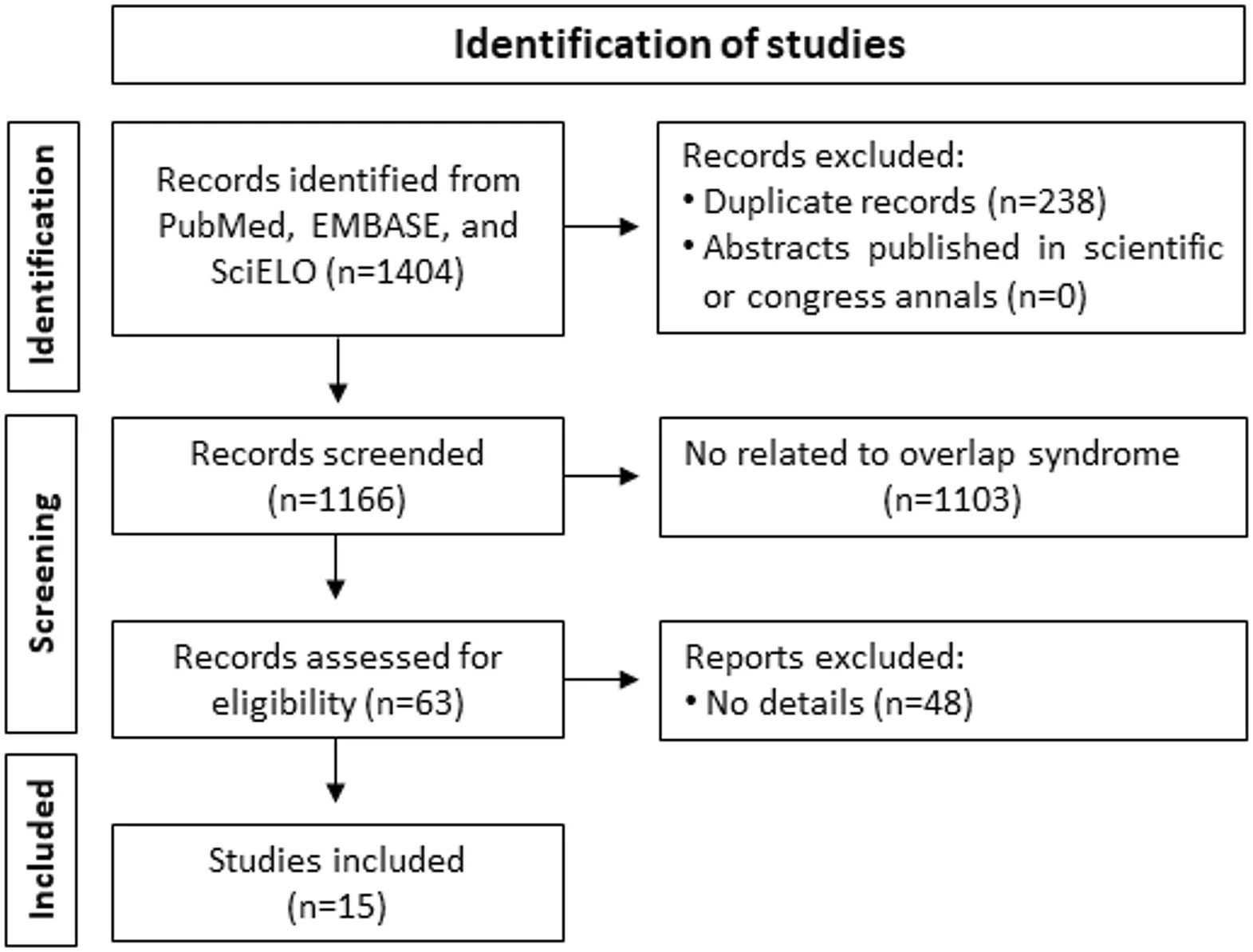
Flowchart from the present study

Initially, 1404 records were identified. Duplicate studies (*n* = 238), studies with DMPs (*n* = 1103), and studies lacking information or overlapping mimicry (*n* = 63) were excluded (Fig. 2). Therefore, only 15 studies were included in this review.

Among the 15 articles analyzed, 17 case reports were described: 52.9% of the patients presented with DM, whereas 35.2%, 5.8%, and 5.8% presented with juvenile DM, clinically amyopathic juvenile DM, and CADM, respectively. Approximately two-thirds of the patients were female and of different ethnicities (Table 1).

The average age at diagnosis of DM was 32 (2–59) years, whereas that at diagnosis of psoriasis was 31 (7–63) years.

Regarding the characteristics of DM in the cases (Table 2), 82% of the patients presented with Gottron’ papules and/or heliotrope rash.

Regarding laboratory analysis, three cases [11, 12, 19] presented CPK levels greater than 200 U/L, with the presence of 9222 U/L in the case [19]. From the studies that presented autoantibodies, 60% presented positive antinuclear antibody. Regarding specific-myositis autoantibodies, one-quarter were positive for anti-Jo-1 or anti-Mi-2.

Triggers for the disease were infrequent, with worsening condition in cases [7] with the use of adalimumab and [18] with the use of secukinumab.

Glucocorticoids are the most commonly used treatments for psoriasis. Only one case [16] reported exacerbation of the disease with the use of ultraviolet radiation. The primary patient characteristics are shown in Table 3.

Among the related comorbidities, one case [9] presented with concomitant non-alcoholic steatohepatitis, whereas in one case [15], the patient had Sjögren’s disease and Hashimoto’s thyroiditis. In one case [18], the patient was diagnosed with diabetes mellitus, systemic arterial hypertension, and glomerulonephritis. Finally, one case [19] was diagnosed with renal failure, palmoplantar keratoderma, and staphylococcal skin lesions and received palliative care due to the appearance of liver neoplasia.

## Discussion

The coexistence of DMPs was infrequent in our sample (2.7%), with 1.5% and 7.6% specifically in patients with DM and CADM, respectively. The literature also underscores the rarity of this condition, reporting only 17 cases over a period of 74 years (1950–2024). Our data revealed a discrepancy in patient profiles when compared with the literature. Despite the predominance of females in both groups, most DM cases were characterized as CADM in our study, whereas DM and juvenile DM were predominant in the literature. Furthermore, the age range in relation to both DM and psoriasis diagnosis was higher in our study, while the literature included pediatric cases. In the present study, DM was defined according to both the Bohen and Peter [21] and EULAR/ACR 2017 criteria for idiopathic inflammatory myopathies [22]. CADM was also defined according to Gerami et al.’s criteria [23]. The cases reported in the literature exhibited variability in terms of diagnostic criteria; three studies affirmed that they followed the aforementioned guidelines [6, 10, 18], whereas others considered, for instance, the presence of “mechanic’s hands”” and laboratory analysis as DM criteria [9, 13]. Regarding the diagnosis of psoriasis, the report of the specific location of the lesions and the presence of biopsy were provided by the tertiary center; however, in the literature, cases [6–8, 13, 18, 19] did not report the types of lesions and their locations, which are crucial factors for diagnosis. In both cases, concomitant diseases are uncommon, and the diagnosis is often complex, considering the variety of myositis that resemble each other and can lead to different diagnoses [25]. Additionally, evidence in the literature demonstrates that patients with DM can exhibit lesions resembling those associated with psoriasis, potentially leading to erroneous diagnosis and, consequently, inadequate follow-up of the patient’s condition [26, 27]. However, the primary presence of psoriasis can complicate the diagnosis of DM because initial examinations may indicate recurrence, which leads to the exclusion of DM, as in the case of Williams et al. [28], in which the diagnosis of DM was delayed and the patient’s clinical condition deteriorated.

In general, the research presented significant data for patient follow-up, such as the interval between disease onset, which has not been reported in many cases. Furthermore, it is recognized that specific and nonspecific autoantibody markers for DM are relevant for excluding other diagnoses. Consequently, our center analyzed all patients with specific and nonspecific myositis autoantibodies for myositis. In contrast, cases in the literature did not report such data [8, 11, 17, 20]. Laboratory tests for CPK and aldolase levels, which are crucial for monitoring the progression of DM, were presented in all patients; however, in some studies in the literature, this was not reported [8, 13, 17, 18, 20]. Conversely, both in this investigation and in the published cases [6, 8, 9, 11, 13, 15–17], treatment primarily consisted of methotrexate, corresponding to its indication presented in the literature [27]. In the present study, despite the presence of diagnostic criteria for concomitant diseases, due to the limited number of cases, we were unable to demonstrate the influence of both pathological conditions in relation to the patient’s illness, as we did not report the impact of DM and psoriasis on each other and on other comorbidities.

## Conclusions

This study identified cases of DMP in our samples, with a median diagnosis age of 38 years for DM and 43 years for psoriasis. However, variability in the diagnostic criteria remains a concern, underscoring the need for standardized approaches to enhance patient management. Overall, this study provides valuable insight into this uncommon overlap.

## Data Availability

No datasets were generated or analysed during the current study.

## Abbreviations

ACR: American college of rheumatology
CADM: Clinically amyopathic dermatomyositis
CPK: Creatine phosphokinase
DM: Dermatomyositis
EULAR: European league against rheumatism

## References

[1] Kronzer VL, Kimbrough BA, Crowson CS, Davis JM 3rd, Holmqvist M, Ernste FC. Incidence, prevalence, and mortality of dermatomyositis: a population-based cohort study. Arthritis Care Res (Hoboken). 2023;75:348–55. 10.1002/acr.24786. 34549549 10.1002/acr.24786 PMC8934743

[2] Osman M, Martins KJB, Wong KO, Vu K, Guigue A, Cohen Tervaert JW, et al. Incidence and prevalence, and medication use among adults living with dermatomyositis: an Alberta, Canada population-based cohort study. Sci Rep. 2023;13:16444. 10.1038/s41598-023-43880-7. 37777591 10.1038/s41598-023-43880-7 PMC10542346

[3] Dalakas MC, Hohlfeld R. Polymyositis and dermatomyositis. Lancet. 2003;20:362:971–82. 10.1016/S0140-6736(03)14368-1 14511932

[4] Griffiths CE, Barker JN. Pathogenesis and clinical features of psoriasis. Lancet. 2007;370:263–71. 17658397 10.1016/S0140-6736(07)61128-3

[5] Rendon A, Schäkel K. Psoriasis pathogenesis and treatment. Int J Mol Sci. 2019;20:1475. 10.3390/ijms20061475. 10.3390/ijms20061475 PMC6471628 30909615

[6] Chu D, Yang W, Niu J. Concurrence of dermatomyositis and psoriasis: a case report and literature review. Front Immunol. 2024;15:1345646. 10.3389/fimmu.2024.1345646. 38348029 10.3389/fimmu.2024.1345646 PMC10859436

[7] Xu Z, Xu B, Zhao B, Gao H, Ye S, Wu J. Treatment of refractory psoriasis with dermatomyositis using Upadacitinib. Clin Exp Rheumatol. 2024;42:458–9. 10.55563/clinexprheumatol/ki4jbt. 38153132 10.55563/clinexprheumatol/ki4jbt

[8] Perna DL, Callen JP, Schadt CR. Association of treatment with Secukinumab with exacerbation of dermatomyositis in a patient with psoriasis. JAMA Dermatol. 2022;158:454–6. 10.1001/jamadermatol.2021.6011. 35171205 10.1001/jamadermatol.2021.6011

[9] Schreiber C, Khamlong M, Raza N, Huynh BQ. Overlap of psoriatic arthritis and dermatomyositis. J Investig Med High Impact Case Rep. 2021;9:23247096211057702. 10.1177/23247096211057702. 34911389 10.1177/23247096211057702 PMC8721678

[10] Markovic A, Schuster V, Simon JC, Kunz M. Erythematous scaling lesions of the face, dorsal fingers, elbows, and knees together with symmetrical muscle weakness in a child. Clin Case Rep. 2019;7:1347–9. 10.1002/ccr3.2219. 31360483 10.1002/ccr3.2219 PMC6637353

[11] Xing Y, Xie J, Jiang S, Upasana M, Song J. Co-existence of juvenile dermatomyositis and psoriasis vulgaris with fungal infection: A case report and literature review. J Cosmet Dermatol. 2019;18:1560–3. 10.1111/jocd.12869. 30697901 10.1111/jocd.12869

[12] Kato Y, Yamamoto T. Development of psoriasis with relapse of dermatomyositis-associated interstitial lung disease. Int J Rheum Dis. 2017;20:660–1. 10.1111/1756-185X.13084. 28397348 10.1111/1756-185X.13084

[13] Inkeles MS, No D, Wu JJ. Clinical improvement of a patient with both amyopathic dermatomyositis and psoriasis following treatment with cyclosporine. Dermatol Online J. 2017;23:13030/qt5qh0n741. 29469754

[14] Montoya CL, Gonzalez ML, Ospina FE, Tobón GJ. A rare case of amyopathic juvenile dermatomyositis associated with psoriasis successfully treated with ustekinumab. J Clin Rheumatol. 2017;23:129–30. 10.1097/RHU.0000000000000430. 28099216 10.1097/RHU.0000000000000430

[15] Akiyama M, Ueno T, Kanzaki A, Kuwana M, Nagao M, Saeki H. Association of psoriasis with Hashimoto’s thyroiditis, Sjögren’s syndrome and dermatomyositis. J Dermatol. 2016;43:711–2. 10.1111/1346-8138.13265. 26778723 10.1111/1346-8138.13265

[16] Dicaro D, Bowen C, Dalton SR. Dermatomyositis associated with anti-tumor necrosis factor therapy in a patient with psoriasis. J Am Acad Dermatol. 2014;70:e64–65. 10.1016/j.jaad.2013.11.012. 24528921 10.1016/j.jaad.2013.11.012

[17] Kim NN, Lio PA, Morgan GA, Jarvis JN, Pachman LM. Double trouble: therapeutic challenges in patients with both juvenile dermatomyositis and psoriasis. Arch Dermatol. 2011;147:831–5. 10.1001/archdermatol.2011.49. 21422326 10.1001/archdermatol.2011.49

[18] Machado NP, Camargo CZ, Oliveira AC, Buosi AL, Pucinelli ML, Souza AW. Association of anti-glomerular basement membrane antibody disease with dermatomyositis and psoriasis: case report. Sao Paulo Med J. 2010;128:306–8. 10.1590/s1516-31802010000500012. 21181073 10.1590/S1516-31802010000500012 PMC10948060

[19] Gran JT, Gunnarsson R, Mørk NJ. En Mann med Erytrodermi, Muskelsvakhet Og vekttap [A man with erythrodermia, muscle weakness and weight loss]. Tidsskr nor Laegeforen. 2009;129:2240–1. 10.4045/tidsskr.09.0184. 19898573 10.4045/tidsskr.09.0184

[20] Pavlović MD, Zecević RD, Zolotarevski L. Psoriasis in a patient with dermatomyositis. Vojnosanit Pregl. 2004;61:557–9. 10.2298/vsp0405557. 15551809 10.2298/vsp0405557p

[21] Bohan A, Peter J. Polymyositis and dermatomyositis. N Engl J Med. 1975;292:344–7. 10.1056/NEJM197502202920807. 1090839 10.1056/NEJM197502132920706

[22] Lundberg IE, Tjärnlund A, Bottai M, Werth VP, Pilkington C, Visser M, International Myositis Classification Criteria Project consortium, The Euromyositis register and The Juvenile Dermatomyositis Cohort Biomarker Study and Repository (JDRG) (UK and Ireland), et al. 2017 European league against rheumatism/american college of rheumatology classification criteria for adult and juvenile idiopathic inflammatory myopathies and their major subgroups. Ann Rheum Dis. 2017;76:1955–64. 29079590 10.1136/annrheumdis-2017-211468 PMC5736307

[23] Gerami P, Schope JM, McDonald L, Walling HW, Sontheimer RD. A systematic review of adult-onset clinically amyopathic dermatomyositis (dermatomyositis siné myositis): a missing link within the spectrum of the idiopathic inflammatory myopathies. J Am Acad Dermatol. 2006;54(4):597–613. Epub 2006 Jan 23. PMID: 16546580. 16546580 10.1016/j.jaad.2005.10.041

[24] Cruellas MG, Viana Vdos S, Levy-Neto M, Souza FH, Shinjo SK. Myositis-specific and myositis-associated autoantibody profiles and their clinical associations in a large series of patients with polymyositis and dermatomyositis. Clin (Sao Paulo). 2013;68(7):909–14. 10.6061/clinics/2013(07)04. PMID: 23917652; PMCID: PMC3715024. 10.6061/clinics/2013(07)04 PMC3715024 23917652

[25] Baer AN. Differential diagnosis of idiopathic inflammatory myopathies. Curr Rheumatol Rep. 2006;8:178–187. 10.1007/s11926-996-0023-5. PMID: 16901075. 10.1007/s11926-996-0023-5 16901075

[26] Cabezas-Rodríguez I, Morante-Bolado I, Brandy-García A, Queiro-Silva R, Mozo L, Ballina-García FJ. Anti-MDA5 dermatomyositis mimicking psoriatic arthritis. Reumatol Clin (Engl Ed). 2018;14:224–6. 10.1016/j.reuma.2016.10.010. English, Spanish. 28040421 10.1016/j.reuma.2016.10.010

[27] Haroon M, Devlin J. Gottrons’s papule in amyopathic dermatomyositis mimicking psoriasis. Clin Rheumatol. 2009;28:1245–6. 10.1007/s10067-009-1244-6. 19655085 10.1007/s10067-009-1244-6

[28] Williams LH, Raugi GJ, Dhaliwal G, Saint S, Lipsky BA. Are we there yet? J Hosp Med. 2007;2:181–8. 10.1002/jhm.210. 17551950 10.1002/jhm.210

